# An Atypical Presentation of Fibrodysplasia Ossificans Progressiva and the Imperative for Multidisciplinary Care: A Case Report

**DOI:** 10.7759/cureus.43309

**Published:** 2023-08-10

**Authors:** Shahzeb Saeed, Husnain Naveed, Niloufar Maktabijahromi, Norhan Mohammed, Abdur Rehman

**Affiliations:** 1 Internal Medicine, Army Medical College, Rawalpindi, PAK; 2 Internal Medicine, Shifa Tameer-E-Millat University Shifa College of Medicine, Islamabad, PAK; 3 Family Medicine, St. George's University School of Medicine, St. George, GRD; 4 Pediatrics, St. George's University School of Medicine, St. George, GRD; 5 Surgery, Mayo Hospital, Lahore, PAK

**Keywords:** bone morphogenetic protein (bmp) signaling, acvr1 gene mutation, genetic disorder, heterotopic ossification, fibrodysplasia ossificans progressiva

## Abstract

Fibrodysplasia ossificans progressiva (FOP) is a rare genetic disorder characterized by the gradual heterotopic ossification of soft tissues, leading to abnormal bone growth within muscles, tendons, and ligaments, due to a mutation in the ACVR1 gene. This specific case report highlights an unusual occurrence of FOP, emphasizing the diagnostic challenges and the importance of quick identification and appropriate intervention to mitigate its debilitating effects. The report also underscores the need for comprehensive genetic counseling and a multidisciplinary treatment approach, involving experts, such as orthopedic specialists, geneticists, and physical therapists, to improve the prognosis and overall well-being of those affected by FOP.

## Introduction

Fibrodysplasia ossificans progressiva (FOP) is a rare genetic disorder causing heterotopic ossification of soft tissues, leading to abnormal bone growth in muscles, tendons, and ligaments. With a global incidence of one in two million, it is one of the rarest genetic diseases, first identified in the 17th century and classified in 1968 [1]. FOP is linked to a mutation in the ACVR1 gene, affecting bone morphogenetic protein (BMP) signaling and causing abnormal bone formation. Current research is investigating the mechanisms behind this, with evidence suggesting inflammation and tissue damage as triggers [2].

Clinically, FOP emerges in childhood, forming heterotopic bone in areas like the axial skeleton and proximal extremities. Early symptoms can lead to misdiagnosis, but over time, swellings become rigid bony masses, limiting mobility and causing disability [3]. Diagnosis involves clinical indicators, radiographic techniques, and genetic testing for the ACVR1 mutation, although testing may not always be accessible [4].

Treatment for FOP is supportive, focusing on preventing trauma, maintaining mobility, and managing pain and inflammation. Surgery is generally avoided. Collaborative care involving specialists and genetic counseling is essential [5]. This case report highlights an unconventional FOP occurrence, diagnostic challenges, and the importance of prompt management, genetic counseling, and ongoing research to improve outcomes and provide hope for those affected by this complex disorder.

## Case presentation

A 16-year-old female patient sought care at an orthopedic clinic, presenting with symptoms of incrementally worsening joint stiffness and limited range of motion in her left shoulder and right hip. These symptoms began to surface three years prior as mild discomfort and sporadic stiffness following physical exertion. Over time, however, the symptoms escalated in severity and persistence, markedly inhibiting her daily activities and overall mobility.

The patient's medical history was devoid of significant events, and there were no incidents of trauma to the affected joints. In addition, she negated any instances of joint swelling or redness, and her family history was equally nonindicative of related symptoms or musculoskeletal conditions. Physical examination exposed the presence of tangible bony nodules within the soft tissues encasing the affected joints. There was no inflammation or erythema in the skin overlying these nodules. The range of motion in both the left shoulder and right hip was acutely constrained, with any attempts at movement eliciting pronounced pain.

**Figure 1:**
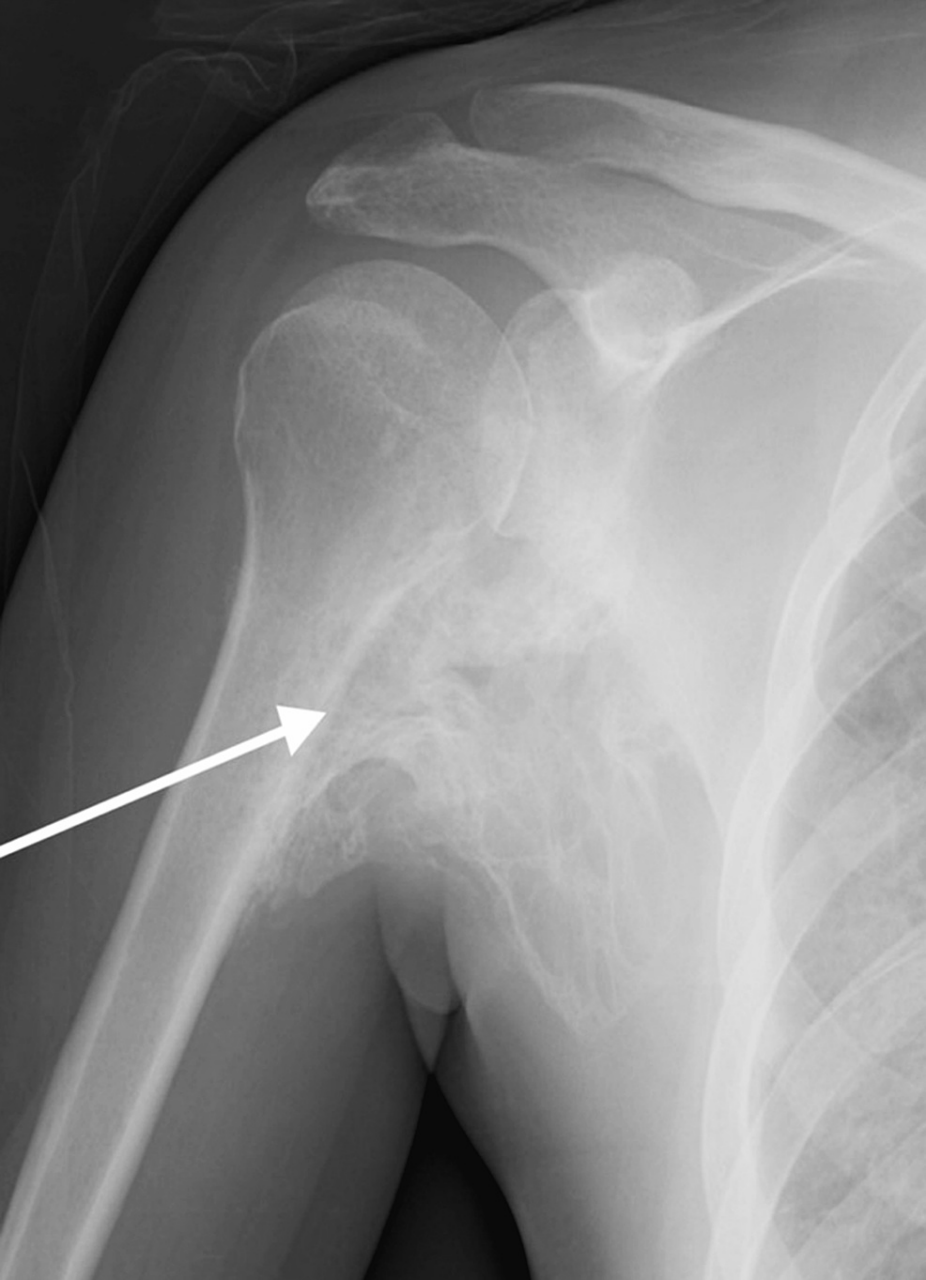
Heterotopic ossification in the right shoulder.

**Figure 2:**
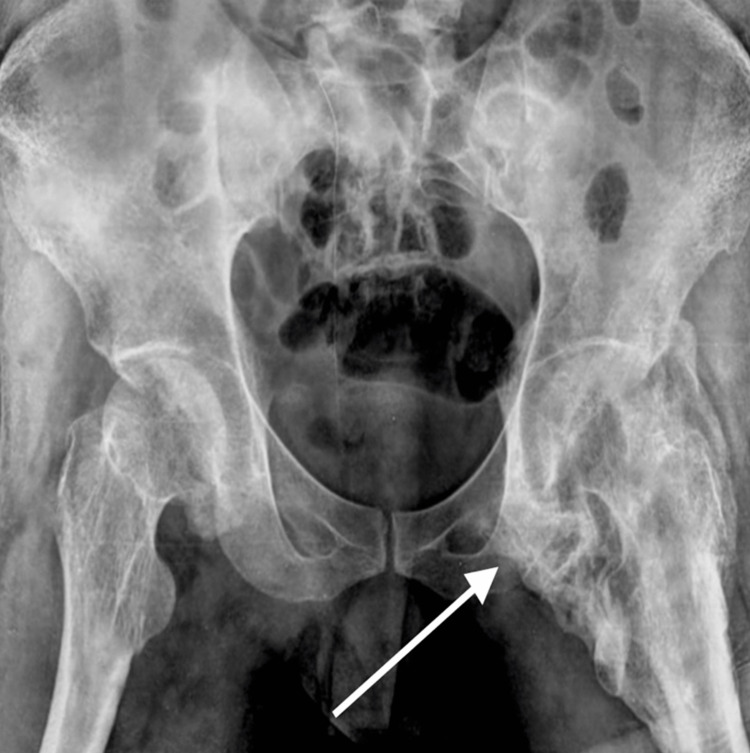
Heterotopic ossification in the hip joint.

Considering the unconventional presentation and the possibility of an underlying genetic condition, further examinations were undertaken. Radiographic analysis, including X-rays, unveiled heterotopic ossification inside the muscles, tendons, and ligaments adjacent to the right shoulder (Figure 1) and hip joints (Figure 2). The bony masses were irregular and appeared fused to neighboring bones, manifesting a characteristic "candle wax dripping" appearance. These diagnostic findings were aligned with the clinical manifestation of FOP.

Genetic testing was conducted, revealing a heterozygous mutation in the ACVR1 gene, specifically the Arg206His variant, thereby corroborating the diagnosis of FOP. Both the patient and her family were counseled about the genetic inheritance pattern associated with FOP, and discussions were held about the potential consequences this might have on future family planning decisions.

To manage her symptoms and decelerate the progression of heterotopic ossification, an integrated care plan was developed, engaging orthopedic specialists, physical therapists, and pain management experts. The patient was guided in the implementation of gentle exercises that promote the range of motion, along with stretching techniques tailored to preserve joint mobility and fend off contractures. The prescription of nonsteroidal anti-inflammatory drugs (NSAIDs) was made to alleviate pain and inflammation during episodes of exacerbation. Regular follow-up appointments were arranged to track the progression of the disease and to address any emergent symptoms or concerns.

Despite this extensive management strategy, the patient's functional limitations continued to escalate over time, reflecting the relentless advancement of heterotopic ossification. Assistive devices, including crutches and a wheelchair, eventually became necessary to support her in performing daily life activities. Given the profound emotional and psychological toll that chronic conditions like FOP can impose, ongoing psychosocial support and counseling were furnished to help her cope with the wide-ranging impacts of the disease on her overall well-being.

## Discussion

FOP is an extraordinarily rare genetic condition, marked by the progressive heterotopic ossification of soft tissues, culminating in the abnormal formation of bone within muscles, tendons, and ligaments. This disorder arises from a mutation in the ACVR1 gene, leading to imbalanced BMP signaling. While the pathogenesis of FOP is well understood, the precise mechanisms that initiate and drive the heterotopic ossification process remain subject of ongoing research [6].

The defining characteristic of FOP is the formation of bony masses within soft tissues, predominantly affecting the axial skeleton, proximal extremities, and connective tissues. The progression of heterotopic ossification typically follows an orderly pattern, beginning with initial nodules or swellings that gradually metamorphose into rigid, immobile bony structures. Oftentimes, the ossification process is catalyzed by tissue injury or inflammation, and factors, such as trauma or surgical interventions, can serve as powerful triggers for the commencement or worsening of ossification [7].

While FOP is characterized by congenital malformation of the great toes and progressive heterotopic ossification, our patient exhibited incrementally worsening joint stiffness and limited range of motion in her left shoulder and right hip without any incidents of trauma to the affected joints or any instances of joint swelling or redness. The absence of clear inflammatory signs and delayed symptom onset is not commonly reported in the literature for FOP patients, making our patient's case particularly intriguing.

FOP is typically inherited in an autosomal dominant manner. This means that an individual with FOP has a 50% chance of passing the condition to each of their offspring. However, the majority of FOP cases arise from de novo mutations, meaning that they occur spontaneously without any previous family history of the condition. In our patient's case, the mutation was also identified as a de novo occurrence. The absence of a family history of FOP often complicates the diagnostic process and can be a source of significant concern for the affected families. In our discussions with the patient and their family, we emphasized the spontaneous nature of this mutation and provided reassurance regarding the low risk of recurrence in future siblings or generations.

The diagnosis of FOP is typically made through a combination of clinical findings, radiographic imaging, and genetic analysis. X-rays and CT scans are essential tools, allowing physicians to observe the extent of heterotopic ossification, often seen as a "candle wax dripping" effect. Genetic testing for the ACVR1 gene mutation confirms the diagnosis and provides insight into the disease's unique genetic basis, potentially informing future therapeutic strategies.

The management of FOP is primarily palliative, focusing on symptom relief, inflammation control, and injury prevention to soft tissues. Care includes physical therapy and stretching exercises to maintain joint mobility and prevent contractures, leading to further disability. While NSAIDs and glucocorticoids may ease inflammation, their ability to halt heterotopic ossification is limited, highlighting the need for ongoing research and targeted interventions.

Surgical interventions in FOP are generally avoided due to the risk of worsening heterotopic ossification. In rare, exceptional cases where life-threatening complications or severe impairments demand surgery, the decision is made with extreme caution. Such instances require meticulous planning, a careful assessment of risks and benefits, and collaboration among a multidisciplinary team. The primary goal is to address immediate medical needs while minimizing potential negative consequences, with the patient's overall well-being as the central focus [4].

FOP profoundly impacts the quality of life, leading to acute disability and significant functional limitations. Its physical effects include restricted mobility, difficulty in daily tasks, and considerable emotional distress. An integrated approach to management is vital, encompassing psychosocial support and counseling to address both physical symptoms and the emotional toll on affected individuals and their families. This comprehensive care enhances the overall well-being of those struggling with this debilitating condition.

In managing FOP, genetic counseling is crucial, providing information, support, and guidance to affected individuals and families. It explains the genetic inheritance pattern, family planning implications, and the unique genetic basis of FOP. This counseling enables informed decisions, aligns personalized medical strategies, and offers support beyond clinical care, contributing to a holistic approach to this rare condition [8].

Current research on FOP focuses on understanding the dysregulated BMP signaling pathway to develop targeted therapeutic interventions. Strategies, such as anti-inflammatory agents, BMP pathway inhibitors, and gene therapy, are being examined in preclinical studies and clinical trials to assess their ability to halt or slow heterotopic ossification. While these innovative approaches offer hope, further investigations are needed to fully evaluate their safety and long-term effectiveness [9].

## Conclusions

This case report highlights an atypical presentation of FOP, characterized by progressive bone formation in soft tissues. The diagnosis was complex due to delayed symptom onset and absence of clear inflammatory signs. Quick identification and careful management are vital to mitigate this rare genetic disorder's debilitating effects. A multidisciplinary approach involving specialists in orthopedics, genetics, physical therapy, and pain management is key to improving patient outcomes. Ongoing research is crucial for understanding FOP's pathogenesis and developing targeted therapies to enhance prognosis and quality of life. Genetic counseling, providing support and guidance to those affected and their families, is a fundamental part of the overall management strategy.
